# Validating Satellite-Derived Land Surface Temperature with *in Situ* Measurements: A Public Health Perspective

**DOI:** 10.1289/ehp.1206176

**Published:** 2013-06-07

**Authors:** Jalonne L. White-Newsome, Shannon J. Brines, Daniel G. Brown, J. Timothy Dvonch, Carina J. Gronlund, Kai Zhang, Evan M. Oswald, Marie S. O’Neill

**Affiliations:** 1 School of Public Health, and; 2 School of Natural Resources and Environment, University of Michigan, Ann Arbor, Michigan, USA; 3 Division of Epidemiology, Human Genetics and Environmental Sciences, School of Public Health, University of Texas Health Science Center at Houston, Houston, Texas, USA; 4 Department of Atmospheric, Oceanic and Space Sciences, and; 5 Risk Science Center, University of Michigan, Ann Arbor, Michigan, USA

**Keywords:** epidemiology, ground truthing, heat, Landsat satellite, land surface temperature, remote sensing, surface imperviousness, temperature, urban areas

## Abstract

Background: Land surface temperature (LST) and percent surface imperviousness (SI), both derived from satellite imagery, have been used to characterize the urban heat island effect, a phenomenon in which urban areas are warmer than non-urban areas.

Objectives: We aimed to assess the correlations between LSTs and SI images with actual temperature readings from a ground-based network of outdoor monitors.

Methods: We evaluated the relationships among *a*) LST calculated from a 2009 summertime satellite image of the Detroit metropolitan region, Michigan; *b*) SI from the 2006 National Land Cover Data Set; and *c*) ground-based temperature measurements monitored during the same time period at 19 residences throughout the Detroit metropolitan region. Associations between these ground-based temperatures and the average LSTs and SI at different radii around the point of the ground-based temperature measurement were evaluated at different time intervals. Spearman correlation coefficients and corresponding *p*-values were calculated.

Results: Satellite-derived LST and SI values were significantly correlated with 24-hr average and August monthly average ground temperatures at all but two of the radii examined (100 m for LST and 0 m for SI). Correlations were also significant for temperatures measured between 0400 and 0500 hours for SI, except at 0 m, but not LST. Statistically significant correlations ranging from 0.49 to 0.91 were observed between LST and SI.

Conclusions: Both SI and LST could be used to better understand spatial variation in heat exposures over longer time frames but are less useful for estimating shorter-term, actual temperature exposures, which can be useful for public health preparedness during extreme heat events.

## Introduction

Use of thermal remote sensing and advanced spatial modeling are emerging trends in environmental epidemiology and public health. Geospatial technologies provide a valuable resource to assist public health practitioners and emergency response planners in identifying areas that are most at risk and using these scientific outputs to inform policies and practices. Thermal remote sensing products such as thermal images captured by the Landsat-5 Thermal Mapper (L5-TM) (NASA 2013) instrument have been used to study areas of higher relative temperatures within a city, also known as micro-urban heat islands (Johnson 2009).

L5-TM has an advantage over other sensors, such as the Moderate Resolution Imaging Spectroradiometer (MODIS) (NASA 2011), in that L5-TM provides a spatial resolution of 120 m (compared with 1,000 m for the thermal band of MODIS); however, it provides only 16-day repeatability, at best, compared with 1-day repeatability for MODIS (Aniello et al. 1995). The Advanced Spaceborne Thermal Emission and Reflection Radiometer (ASTER) is another sensor that could be used, but imagery is not available free of charge (NASA Jet Propulsion Laboratory 2012). The data captured by satellite can be transformed into several helpful measures, including land surface temperatures (LST) and percent surface imperviousness (SI). LSTs are a primary factor in determining surface radiation and human comfort in cities (Weng 2009).

The higher spatial resolution of L5-TM information is important in micro-urban heat island studies, so we have focused on those data here. The SI is defined as the percent of the surface of an area that is not penetrable by water, such as concrete or asphalt, and can be mapped at a 30-m resolution with L5-TM. This characteristic has been commonly used in studies to assess the degree of urbanization of an environment as well as explore the spatial extent of surface urban heat islands (Roy and Yuan 2009).

The relationship between LST and vegetated areas has been documented in the literature. A study by Aniello et al. (1995) compared the spatial distribution of micro-urban heat islands and tree cover in Dallas, Texas, using L5-TM and geographic information systems (GIS). They examined the usefulness of L5-TM for classifying tree-cover information and using thermal band 6 to produce a thermal map of Dallas, Texas. Their methods involved processing and classifying L5-TM images and tree cover data in GIS. Although L5-TM data were useful for mapping micro-urban heat islands in Dallas, the authors recommended use of exact on-the-ground temperatures for image calibration in future studies. Remote sensing data have been used to help model urban surface temperatures; specifically, validating LST data with actual on-the-ground temperature measurements, known as ground-truthing. For example, strong correlations between satellite-derived air temperatures and *in situ* measurements were found when characterizing urban heat island intensity in Hong Kong, using ASTER satellite imagery (Fung et al. 2009). Comparisons between ground temperatures and estimated temperatures using imagery from MODIS, the National Oceanic and Atmospheric Administration Advanced Very High Resolution Radiometer (NOAA-AVHRR), and L5-TM, showed a very high correlation in both urban and rural areas (Rigo et al. 2006). Although previous studies have used Landsat to create prediction models for surface temperature, and shown strong correlations between surface temperature and surface imperviousness (Yuan and Bauer 2007), few studies have simultaneously explored the relationship among SI, ground-based temperature measures, and LST calculated from thermal imagery.

Adaptation to the consequences of climate change, as future scenarios of heat-related morbidity and mortality become a major public health concern, requires predicting areas of high vulnerability to heat in cities. Epidemiologic studies on heat and health have begun to use satellite-derived temperatures (instead of temperature data from the nearest airport) and land cover to potentially provide more refined heat exposure classifications. A study in Philadelphia used GIS and thermal imaging to investigate the relationship between the spatial distributions of vulnerable populations, urban heat island intensities, and heat-related deaths (Johnson and Wilson 2009). The authors recommended that more multi-year studies use spatial modeling and remote sensing methods to better help determine areas of risk throughout cities

Uejio et al. (2011) used ASTER data to determine the magnitude and spatial variation of mean radiant surface temperature for different densities of impervious surface area (ISA), to further document the change in air trends and air quality that can result from transforming land use from rural to urban. Harlan et al. (2013) used Landsat (30-m resolution) to calculate the Normalized Difference Vegetative Index (NDVI) and estimated surface temperatures.

There is still a need to understand how proxies for heat exposure correlate with actual heat exposure. Harlan et al. (2013) provided correlations between the mean NDVIs and surface temperatures at the census block group level. Our study goes beyond this comparison by correlating SI, land surface temperature (estimated from Landsat), and actual temperature measurements made by a ground-based temperature monitor network over the summer. Our study is novel for evaluating the correlations between satellite-derived temperatures and a ground-based temperature network with high temporal (10 min) and spatial resolution (19 monitors over a range of levels of SI within a single county) at a height relevant to human health (1.5 m above the ground). Further, it characterizes these features in metropolitan Detroit, Michigan, where people may be poorly adapted to heat and where several epidemiologic studies have already shown important and socially unequal health consequences associated with hot weather (Anderson and Bell 2009; O’Neill et al. 2003; Schwartz et al. 2004; Sheridan and Kalkstein 2010).

Indeed, integrating information from ground-based temperature monitoring networks and satellite-derived images using a GIS platform can provide useful data for exposure assessments in most urban areas, specifically for the study of heat-related death and illness. Validation of satellite data sources by ground truthing (i.e., information that is collected on location) can help characterize and identify neighborhood-level urban heat islands; this information could be useful for public health professionals and urban planners to prevent heat-related mortality and other adverse health effects from high summer temperatures. Such data could also direct intervention strategies to reduce the urban heat island effect. Some previously published work did not explicitly address the practical challenges of integrating insights from ground-truthing studies with public health research and applications (Lo and Faber 1997).

The purpose of this study is to apply a public health perspective to a determination of whether spatial variation of temperatures within a network of ground-based outdoor temperature monitors is correlated with satellite-derived LST and SI. Although we did not expect LST (which represents the temperature of the ground) to completely predict the temperature of the air at a height relevant to human health, we hypothesized that the air temperatures measured by a ground-based temperature monitoring network would be highly correlated with LST as well as with values of SI.

## Methods

*Ground-based temperature-monitoring network and surface imperviousness.* Sites in our ground-based temperature monitor network in the Detroit metropolitan region (Wayne County, MI) were selected, with site SI values ranging from 0 to 100% imperviousness and with buffer zones around each site ranging from 0 to 800 m. We picked both urban and rural locations to assess the temperature differences among areas within the same county that might have different land-use patterns. This strategy was designed partly to evaluate the existence of urban heat island structure in the Detroit metropolitan region (Oswald et al. 2012; Zhang et al. 2011). Using SI from the U.S. Geologic Survey (USGS) National Land Cover Database (NLCD) product (Multi-Resolution Land Characteristics Consortium 2013), we performed half-mile smoothing of every pixel and classified SI by decile. Once the SI surrounding various prospective residential sites for placing the temperature monitors was established and a range of SI levels was ensured by the sampling strategy, home occupants were approached, told the purpose of the study, and asked if they would be willing to have a temperature monitor outside their homes. All volunteers signed an agreement letter to participate. The research was compliant with all relevant national, state, and local human subjects regulations.

HOBO Pro v2 U23-002 (external temperature/relative humidity) outdoor temperature monitoring devices (Onset HOBO Data Loggers, Pocasset, MA) were calibrated and used to record temperature and relative humidity at 10-min intervals from 13 June to 30 September 2009. The calibration process involved collocating the monitors in a controlled environment along with a temperature probe from the National Institute of Standards and Technology (Gaithersburg, MD) to ensure that the temperatures recorded were within the accuracy range reported in the operation manual: ± 0.21^o^C, within an ambient temperature range of 0–50^o^C (Onset HOBO Data Loggers 2013). Monitors were positioned in residential grass-covered backyards of volunteers following a strict placement protocol that required monitors to be sited *a*) at least 10 ft (3 m) away from buildings, homes, and trees; *b*) 1.5 m from the actual surface [to better assess the level of exposure that would be experienced by a person of average height and to be consistent with the instrument siting protocols used by the World Meteorological Organization, as well as NOAA’s National Weather Service (World Meteorological Organization 2008)]; *c*) not in the direct pathway of automatic lawn sprinkling systems; *d*) not in a shady area or near falling objects; *e*) away from power lines and swampy damp ground; and *f* ) facing southwest.

*Processing the L5-TM satellite image to derive land surface temperature*. Images of Earth were taken nearly continuously from 1 March 1984 to January 2013 on a 16-day cycle by the L5-TM, which consistently imaged the Detroit Metropolitan Region at about 1205 hours Eastern Daylight Time (EDT) at each pass. L5-TM captured images collected at a 705-km altitude, 185-km swath, 120-m spatial resolution for thermal band data and 30-m resolution for the other spectral bands. The satellite images captured by L5-TM are free and downloadable from the USGS (USGS 2013).

**Figure 1:**
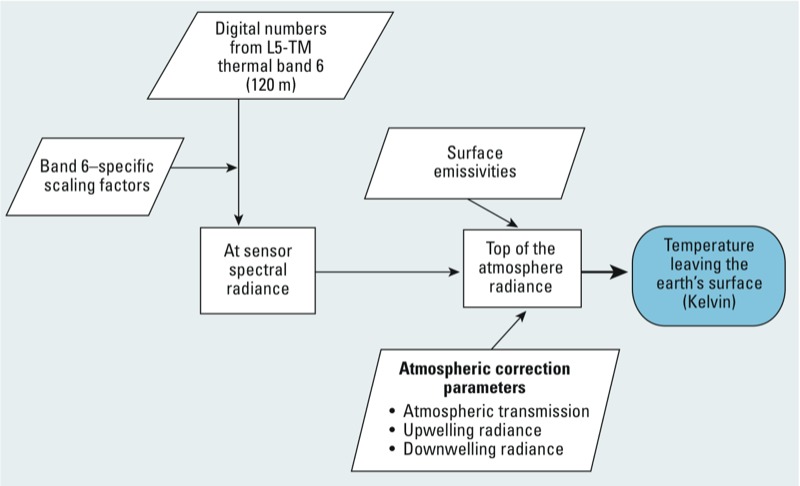
The processing method for converting raw satellite images to land surface temperature. Parallelograms denote data inputs, squares denote calculations, and the shaded oval denotes the final value (temperature leaving Earth’s surface).

Satellite images were downloaded for use only if they met the following criteria: The images were captured during the study period, covered the entire study area (geographically), had < 14% cloud cover, and were taken under clear weather conditions. More cloud cover and unclear weather conditions can inhibit the signal strength reflected back to the satellite and cause underestimation of the ground surface temperature. Each image had seven spectral bands of information. Thermal infrared band 6 (10.4–12.5 μm) provides the data that can be converted from raw digital numbers to LST. To convert from a digital number to a temperature, we needed calibration formulas, atmospheric correction tools, and transformations (Figure 1). Once an image was selected by our criteria, we used ERDAS Imagine 9.2 software (Leica Geosystems, Inc., Atlanta, GA) to convert the image (from a TIFF file to an IMG file) to a usable format for GIS. ArcGIS version 9.3.1 (ESRI, Redlands, CA) was used to perform the calculations outlined below. First we converted the digital numbers taken from the raw image into at-sensor spectral radiance [L_λ_, the temperature read at the sensor, W/m^2^ × sr × μm (watts per square meter per steradian per micrometer)]. The source equations and calibration constants developed specifically for L5-TM images were used from the process outlined by Chander et al. (2009). L_TOA_ was then used to calculate an actual ground surface temperature.

Because the satellite signal received by the sensor is in space, the effects of the atmosphere (i.e., air pollution, weather) and surface emissivity (the ratio of the radiation emitted by a surface to the radiation emitted by a blackbody at the same temperature: i.e., how well different surfaces reflect solar energy) can have considerable influence on the accuracy of the satellite-derived surface temperature. To account for these influences, we used a tool that estimates atmospheric influences in conjunction with a data layer of emissivity for the study area. Using this web-based atmospheric correction parameter tool developed by Barsi et al. (2003), we estimated three scene-specific parameters for each satellite image. The tool required the following inputs for each satellite scene: year, month, day, Greenwich Mean Time (GMT), and latitude and longitude coordinates. The outputs of this tool were the three parameters: atmospheric transmission (T, unitless), upwelling radiance (L_u_, W/m^2^ × sr × µm) and downwelling radiance (L_d_, W/m^2^ ×⊇sr × μm), where W/m^2^ × sr × μm are the units of spectral radiance.

Values of emissivity for the study area were then estimated by examining the land use/land cover designations, downloaded for the region from the 2006 NLCD (Multi-Resolution Land Characteristics Consortium 2013). We considered the differences in emissivity of various impervious surface land cover types in the main equation to calculate surface temperature. Because our land cover data do not distinguish among different types of impervious surface, we were unable to represent possible differences in emissivities among them. We created a layer of emissivity (ε; range, 0–1) for the study area based on reference emissivity values for various land cover classes used in other studies (Lillesand et al. 2007).These values of emissivity, coupled with the atmospheric correction parameters, were used in the following equation to calculate the radiance of a blackbody target of kinetic temperature (L_T_), which ultimately represented surface temperature:

L_T_ = (L_λ_ – L_u_ – (1 – ε) × L_d_)/(Τ × ε), [1]

where L_T_ = radiance of a blackbody target of kinetic temperature and L_TOA_ = at sensor spectral radiance, W/m2 × sr × μm. We then transformed L_T_ into a temperature in Kelvin (using Planck’s equation), and then converted Kelvin to degrees Celsius.

Once the scene was transformed to a surface temperature in units of degrees Celsius, temperatures outside our range of interest (< 0°C) or areas of no data (water) were masked out of the layer (given a value of NoData). The implausible ranges were likely a result of some cloud cover over a certain point, values over a body of water, or possibly a source of error in the reflectance value that would cause noise in the analysis.

*Geographical and statistical analysis*. Using spatial analysis tools in ArcGIS, we averaged the LST and SI over the areas of the following seven concentric circles with different radii around each outdoor monitoring unit (buffers): at the point (0 m) and at 100, 200, 300, 400, 500, and 800 m. We assessed the values at different buffers because they can represent physical processes that can occur at different spatial scales within the urban canopy layer—the layer of the urban atmosphere extending upward from the surface to building height (Roy and Yuan 2009). These spatial scales range from the microlevel (at the home = 0 m) to more macrolevel (block, neighborhood) exposures. The physical mechanism of correlation is that thermometers “sense” temperature that is transferred from the “source area” (i.e., surfaces below, around) to the sensors through turbulent transport. Thus the relationship between source area (i.e., LST) and thermometer depends on both atmospheric and surface states. Furthermore, LST and ground-based temperature can be influenced by a number of physical factors relevant to the study area—surface heterogeneity, considerable variability in temperature over small areas, and physical structures—and the varying buffers allowed exploration of on what scale these factors might operate. From a health perspective, understanding correlations at these different spatial scales can inform tools for use at the urban-local scale, to predict “hot spots” where prevention of heat illness and deaths is especially needed. The grid cells for SI (30-m resolution) and LST (120-m resolution) that were contained completely within and intersected the corresponding buffer were included in the calculation. The zonal-stats operation was used to generate the average LST and SI for each of the buffers. Spearman rank correlation coefficients were calculated between the mean LSTs and SIs and the temperatures from the outdoor monitoring network averaged over five time periods: 1205 hours [to correspond to the time of the satellite image (average of 1200 and 1210 hours)]; average temperature from 0920 to 1210 hours (3-hr average); average temperature from 0400 to 0500 hours on 19 August 2009 (nighttime temperature); average temperature from 1210 hours on 18 August to 1210 hours 19 August 2009 (daily); and, August average monthly temperature (monthly). The maximum and minimum temperatures and standard deviation for each group of measurements were calculated. These different time periods were chosen to see whether LST and/or SI taken at one point in time would give a better picture of instantaneous versus longer-term spatial variation in temperatures in the study area.

Related to this point, we explored how well the LST captured by the one usable 2009 LST image represented the LST over a longer time frame, especially in this region where population growth and economic development have been static or declining in recent years. To better understand whether the 2009 scene showed a spatial variation of temperatures within the region similar to other images from different time points and different years, we processed and evaluated multiple LST images from a 10-year time span in the same manner as the 2009 image. From 1999 to 2009, we found only four “high quality” images (for 2002, 2003, 2004, and 2008) during our study months of 1 June–31 August. Correlation coefficients were calculated comparing a composite image (i.e., all five usable LST scenes overlaid in GIS) versus each single-year scene. Additionally, the entire LST scenes (i.e., all 245,188 pixels, 120-m resolution) were used for this analysis, not just the temperatures extracted at the various buffers around the HOBO monitors.

SAS version 9.2 (SAS Institute Inc., Cary, NC) was used for all statistical analysis, and ArcGIS 9 was used for all spatial analysis.

## Results

**Figure 2:**
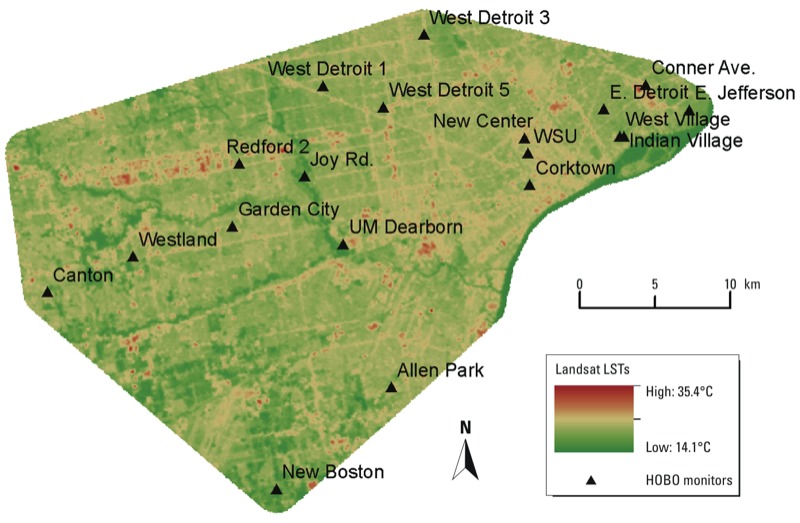
Final processed L5-TM image of the Detroit metropolitan region study area and the locations of the ground-based temperature monitors (HOBOs). Abbreviations: UM, University of Michigan; WSU, Wayne State University.

Temperature data from 19 of 24 ground-based monitors positioned throughout the county were used in the analysis. Five of the temperature monitors were excluded due to siting conditions that could have jeopardized the readings, such as being too close to a building. A search in the Landsat 4 and L5-TM archive data sets for the Detroit area (latitude 42.331427, longitude –83.0457538), yielded 21 satellite images. Of these 17 had a cloud cover percentage > 14%, and 16 did not cover the study area geographically. Consequently, one image, taken by the satellite on 19 August 2009, met our criteria (i.e., covered the geographic area of interest; had cloud cover of < 14%, captured during the time period of the study (13 June–30 September 2009). This image was acquired during the daytime at 1805 hours GMT (1205 hours EDT). The quality of band 6 was scored as a 9, the highest score for images. This value reflects the quality and level of errors detected in the image (see USGS 2013 for explanation). Figure 2 shows the final processed band 6 image of the study area.

The highest levels of mean SI were seen in the 500-m buffer zones across all of the locations (Table 1). Overall, the New Center location had the highest SIs (range, 42–87.7%), and the New Boston location had the lowest overall SIs (range, 9.0–18.5%). The New Center area location had the highest LSTs compared with other locations, from 24.4 to 25.6°C, whereas the New Boston area had the lowest overall LSTs, from 18.2 to 18.4°C. In terms of the ground-based temperature readings, the instantaneous 1205-hours time point at each location showed a higher maximum temperature than the other recorded time points.

**Table 1:** Summary of satellite-derived average LST measurements and ground-based temperature monitoring network temperatures.

Location name	Percent surface imperviousness at different radii (m) around the outdoor monitoring point						Landsat-derived surface temperatures (°C) at different radii (m) around the outdoor monitoring point						Ground-based temperature network readings (°C)				
	0	100	300	400	500	800	0	100	300	400	500	800	Sat^*a*^	3-hr avg^*b*^	Night^*c*^	Daily avg^*d*^	Monthly avg^*e*^
Allen Park	13.0	29.3	51.5	50.9	47.9	44.8	21.6	21.3	22.2	22.3	22.3	21.8	26.5	25.3	19.6	24.0	21.7
Canton	59.0	33.8	36.3	36.2	37.1	35.5	21.1	21.4	21.8	22.0	21.9	21.7	25.6	24.4	17.1	22.2	20.7
Conner	69.0	64.1	75.1	74.3	74.8	73.4	23.4	23.1	22.7	23.0	23.1	23.7	26.3	25.2	18.8	24.4	22.0
Corktown	35.0	48.9	48.8	53.0	59.7	65.8	21.1	21.6	22.1	22.4	22.9	23.4	27.6	25.6	19.0	23.7	21.7
East Detroit	61.0	71.0	50.4	52.2	53.4	54.5	22.1	21.8	21.2	21.3	21.3	21.4	26.2	24.9	20.4	24.4	22.0
East Jefferson	32.0	37.0	41.1	44.5	45.1	49.1	19.2	19.2	19.7	20.0	20.1	20.4	23.5	23.4	19.3	23.2	20.9
Garden City	47.0	50.5	50.8	51.1	51.9	52.7	22.0	22.0	21.8	22.0	22.2	22.3	26.7	23.9	18.8	23.4	21.3
Indian Village	58.0	42.2	41.2	43.5	47.0	51.2	20.4	20.3	20.1	20.4	20.7	21.2	26.7	23.3	19.8	23.8	21.7
Joy Rd.	35.0	19.6	15.1	13.3	12.2	13.7	20.8	21.0	19.6	19.1	18.9	19.0	26.7	25.3	18.2	23.3	21.1
New Boston	9.0	9.0	18.5	18.3	16.8	13.9	18.6	18.4	18.4	18.4	18.4	18.4	27.3	25.5	18.3	22.6	20.9
New Center	42.0	79.2	86.1	87.4	87.7	83.1	24.9	25.6	25.5	25.2	24.9	24.4	26.7	25.1	21.0	24.8	22.3
Redford 2	44.0	49.7	55.7	55.6	56.5	57.0	22.6	22.9	22.8	22.7	22.6	22.4	24.8	24.7	19.3	23.8	21.5
UM Dearborn	14.0	39.1	35.3	37.1	37.5	33.9	21.3	21.2	21.5	21.1	21.1	20.5	26.1	24.8	18.2	23.0	21.1
West Detroit 1	25.0	45.0	43.7	43.3	45.8	51.0	20.8	20.9	21.1	21.2	21.4	21.8	25.1	23.7	19.7	23.6	21.5
West Detroit 3	57.0	66.4	69.6	65.6	62.5	57.3	23.5	23.6	23.3	22.9	22.5	22.2	28.0	24.0	19.7	24.0	21.5
West Detroit 5	55.0	54.4	50.0	53.3	55.8	62.3	21.2	21.1	21.5	21.7	22.0	22.5	27.7	26.4	18.9	23.5	21.6
West Village	30.0	45.0	51.8	50.5	48.1	50.7	20.5	20.5	20.7	20.9	20.9	21.3	26.7	25.3	19.9	24.1	21.7
Westland	84.0	47.4	45.2	47.5	45.3	38.5	24.7	23.9	22.3	22.1	21.7	21.0	26.5	23.2	18.4	23.1	21.2
Wayne State University	32.0	76.1	81.5	75.0	70.9	71.5	23.1	23.7	24.1	23.9	23.6	23.5	26.7	25.3	20.1	24.2	22.0
Minimum	9.0	9.0	15.1	13.3	12.2	13.7	18.6	18.4	18.4	18.4	18.4	18.4	23.5	23.2	17.1	22.2	20.7
Maximum	84.0	79.2	86.1	87.4	87.7	83.1	24.9	25.6	25.5	25.2	24.9	24.4	28.0	26.4	21.0	24.8	22.3
SD	20.1	18.4	18.5	17.9	17.9	18.1	1.7	1.7	1.7	1.6	1.5	1.5	1.1	0.9	0.9	0.6	0.4
Abbreviations: avg, average; UM, University of Michigan.^******^^***a***^Sat: instantaneous satellite temperature taken at 1205 hours. ^***b***^Average temperature from 0920 am to 1210 hours. ^***c***^Average nighttime temperature from 0400 to 0500 hours on 19 August 2009. ^***d***^Average temperature from 1210 hours on 18 August 2009 to 1210 hours 19 August 2009. ^***e***^August average monthly temperature.																	

At least two statistically significant correlations, for each radius distance, were seen between LST and the ground-based temperatures for the daily and monthly temperature for all buffers except 100 m, as shown in Table 2. At least three statistically significant correlations were also seen for each radius distance between SI and ground-based air temperature measurements for nighttime temperature, daily temperature, and monthly temperature at all buffers except the 0-m point. For the relationship between SI and LST, statistically significant correlations were found with Spearman correlation coefficients ranging from 0.49 to 0.91, as shown in Table 3.

**Table 2:** Spearman rank correlation coefficients (*p*-values) between satellite-derived LST and SI measurements, with ground-based temperature measurements.

Measurement	Concentric radii distances (m) around monitoring point used to calculate an average	Ground-based temperature measurements				
		Instantaneous temperature, 1205 hours, 19 August 2009	Average temperature, 0920–1210 hours	Average temperature, 0400–0500 hours, 19 August 2009	Average temperature from 1205 hours, 18 August 2009 to 1210 hours, 19 August 2009	August average monthly temperature
LST	At point (0 m)	0.0018 (0.99)	–0.11 (0.66)	0.22 (0.36)	0.47 (0.043)	0.47 (0.041)
100	0.026 (0.91)	–0.10 (0.68)	0.21 (0.38)	0.44 (0.060)	0.44 (0.053)
300	0.0070 (0.98)	–0.0070 (0.98)	0.28 (0.24)	0.48 (0.038)	0.51 (0.027)
400	0.030 (0.90)	0.084 (0.73)	0.33 (0.17)	0.55 (0.015)	0.59 (0.0084)
500	0.068 (0.78)	0.18 (0.45)	0.32 (0.18)	0.55 (0.014)	0.61 (0.0057)
800	0.13 (0.60)	0.25 (0.30)	0.41 (0.085)	0.62 (0.0048)	0.68 (0.0014)
SI	At point (0 m)	0.058 (0.81)	–0.39 (0.098)	–0.0097 (0.97)	0.17 (0.47)	0.71 (0.48)
100	0.16 (0.50)	0.019 (0.94)	0.59 (0.0075)	0.72 (0.0005)	0.69 (0.001)
300	0.081 (0.74)	0.15 (0.54)	0.66 (0.002)	0.83 (< 0.0001)	0.81 (< 0.0001)
400	0.17 (0.48)	0.20 (0.40)	0.59 (0.0077)	0.76 (0.0001)	0.77 (0.0001)
500	0.22 (0.36)	0.22 (0.36)	0.62 (0.0043)	0.81 (< 0.0001)	0.82 (< 0.0001)
800	0.22 (0.37)	0.17 (0.48)	0.60 (0.0062)	0.75 (0.0002)	0.77 (0.0001)
Spearman correlation statistical test was used to calculate a correlation coefficient; *p* < 0.05.						

**Table 3:** Spearman rank correlation coefficients (*r*_S_) between satellite-derived LST and percent SI

LST vs. SI calculated at the following concentric radii (m)	*r*_S_ (*p*-value)
0 (at the point)	0.49 (0.032)
100	0.74 (0.003)
200	0.74 (< 0.0003)
300	0.79 (< 0.0001)
400	0.84 (< 0.0001)
500	0.86 (< 0.0001)
800	0.91 (< 0.0001)

In the analysis comparing spatial variation in LST using five summertime satellite images from 2002, 2003, 2004, 2008, and 2009, LST temperature ranges and the areas with the highest temperatures were consistent over the years. The 2009 LST scene was highly correlated with the 5-year composite LST scene (*R*^2^ = 0.96). We also found a high correlation between the SI scenes from two different years, 2001 and 2006 (*R*^2^ = 0.98).

## Discussion

The purpose of this study was to assess the relationship between LST and SI measurements and ground-based air temperature measurements in the Detroit metropolitan region. Our results showed a statistically significant relationship between LST and SI at all buffers, as well as LST and SI and the ground-based air temperatures at certain buffers. These correlations between LST and SI are consistent with findings from other published studies (Imhoff et al. 2010; Uejio et al. 2011; Yuan and Bauer 2007; Zhou and Shepherd 2009). This suggests that SI data, which require much less processing than the LST data, could be used as a proxy for LST. Consequently, public health researchers and practitioners may still be able to use a fairly straightforward method to determine city hot spots using high SI as an indicator of potential increased temperature exposure.

Our study used standard methods that facilitate comparisons with other work, and is the first analysis of this kind during the summertime in an large, urban Midwest city. Detroit has unique features, including a higher proportion of vacant lots than in other metropolitan areas. Additionally, our study simultaneously explored the relationship between SI, LST calculated from thermal imagery, and ground-based temperature measures, adding the ground-truthing element that has been called for to independently validate the satellite imagery as a proxy for human-scale exposures (Voogt and Oke 2003).

The consistency of our findings with those of other studies suggests that these unique features do not impair the overall utility of satellite imagery for public health applications. In Detroit, the 2009 satellite-derived LST image—corrected for atmospheric effects and spatial variations in emissivity—as well as the SI image from the 2006 NLCD might be suitable to represent air temperature variability between sites for heat exposure studies in the region or for targeting heat-health interventions. Because our land-cover data do not distinguish among different types of impervious surface, we were unable to represent possible differences in emissivities among them, and this is a type of variability that contributes to possible uncertainties in our analysis. The analysis we did comparing the 2009 scene with LST calculated from four previous years’ summertime scenes showed that the LST estimated from the satellite images was relatively consistent over time, suggesting that changes in land use were not substantial in the Detroit metropolitan region. Further, for application in heat-health studies, LST is better suited for representing physical properties that are stable over time and can affect human temperature exposure rather than as a proxy for actual ambient air temperature at a particular point in time.

Our results complement those of two sister studies of the Detroit metropolitan region that examined spatial variation of temperature during the entire summer of 2008. The first study used the same observational network of air temperature monitors that we used in the present study in conjunction with airport temperature monitors and monitors operated by the state of Michigan Department of Environmental Quality (Oswald et al. 2012). This study found the correlation between summer mean daily low temperature anomalies (the daily residuals at each location minus measurement uncertainty) in 2009 and SI in 2001 (*r* = 0.68 at the 200-m buffer, *p* < 0.001) to be higher than between daily temperature anomalies and other geographic characteristics. This suggests that in relatively sprawling cities, the urban heat island most closely follows SI and would have a unique structure in each city based on the SI structure (Oswald et al. 2012). A second study used geospatial approaches to create a continuous, spatial layer of estimated air temperature, and found high correlations between SI measured in 2006 and observed air temperatures in 2008 (Zhang et al. 2011). Neither of those two studies examined LST, but the fact that they observed correlations between SI and air temperatures using different methodologies supports our finding that SI and, by extension, LST are moderately correlated with air temperature.

Previous studies have ground-truthed L5-TM data using airborne thermal scanner flights (Voogt and Oke 1998) or using satellite data in conjunction with ground-based air temperature measurements with other remote sensing predictors to create a model for air temperature (Cristóbal et al. 2008). Other researchers have also created a spatiotemporal general linear model to estimate surface temperature using several predictors with 15 Landsat multispectral images taken between 1987 and 2002 for the Quebec Province, Canada, spanning June–August (Kestens et al. 2011). Using ambient temperatures recorded from multiple meteorological stations, they found that the 3-day average air temperature was a strong predictor of LST, as were the NDVI and land cover categories. Their results suggest that increasing the number of meteorological and geographical predictors could provide more precise estimates of heat exposure in urban areas.

An added benefit of using SI as a proxy for temperature exposure is that increasing vegetative cover and other changes can reduce the heat-trapping potential of the urban landscape; therefore, results of studies using SI could be of direct relevance for policy changes. This finding might be helpful in the urban planning sector.

Correlations between LST and ground-based temperature measurements (Table 2), were stronger at the largest radii (e.g., 500 m and 800 m), and stronger using the average temperature from day 1 to day 2 and the monthly temperature. Several possible reasons for this come to mind. Urban areas are heterogeneous in topography, physical structures, land use, and the like, so averaging of the LST temperature over a larger buffer zone may drown out the physical noise that can influence the air temperature at a specific point.

The stronger correlations when temperatures are averaged over longer time spans suggest that instantaneous temperatures are less indicative of the overall spatial pattern of the temperature tendencies. Morning temperatures are likely less correlated due to lack of turbulent transport (the main mechanism relating source area to sensor) and influence of cold air drainage (i.e., topography). However, in terms of estimating personal exposure, lower correlation between these satellite data sources and the actual ground-based air temperature readings at 1205 hours and the 3-hour average underscores the importance of identifying other tools that can better gauge actual short-term temperature exposure near the ground surface, especially when health outcomes that can result from acute exposures are of interest. Previous epidemiologic studies of heat and daily mortality that have included Detroit have found that heat exposure on days 0–1 have been most relevant (e.g., Anderson and Bell 2009), although heat wave durations of at least 4 days may have an additional effect (e.g., Gasparrini and Armstrong 2010). The day is the common time unit of analysis for administrative databases of health outcomes (hospitalization, deaths, births), but other clinical outcomes that could be affected by heat (e.g., blood pressure, pulse rate) may be available at a finer time scale, such that hour-specific temperature data would be relevant. Longer duration of warm temperatures could also be relevant to both the exposure and the health resilience of residents, relating to air conditioning use and overall energy demand in homes.

Using the LST and SI data in conjunction with health outcome data could provide a more general understanding of spatial heat vulnerability. For example, an epidemiologic investigation of a 1993 extreme heat event in Philadelphia used satellite imagery and geostatistical methods to determine whether vulnerability to heat-related mortality was higher in areas with higher urban heat intensity (Johnson and Wilson 2009). The authors found that the heat load of the environment detected by the Landsat satellite data was potentially a contributing factor to heat-related deaths during the summer of 1993, and that the thermal data used in this study could be used to develop models of place-based vulnerability.

More frequent daily observations are made by MODIS. However, this sensor records thermal emission at a spatial resolution (1 km) too coarse for micro-urban heat island investigations. Future studies should investigate the public health implications of this trade-off between temporal frequency and spatial resolution. Additionally, researchers have created new methods to better use satellite imagery to assess land surface temperature. In particular, physical and statistical methods for downscaling MODIS scenes (Liu and Pu 2008) and enhanced physical methods that will reduce downscaling uncertainty, reduce the smooth effects, and block effects due to isothermal assumption (Liu and Zhu 2012) could be incorporated into health studies.

Our ground-based temperature monitors were mounted 1.5 m above the ground, and the non-statistically significant relationships that we found between LST and the ground-based temperature monitoring network—for all buffers for the instantaneous and 3-hr average temperatures as well as the 0- to 700-m buffers for average daily temperature—might be a result of mixing, advection, and convection processes within the boundary layer that influence the air temperatures recorded by the outdoor temperature monitor. Because we are comparing two different types of measurements—surface temperature and air temperature—the correlations between these measurements might not be as strong due to logistical (e.g., timing and resolution) as well as physical (e.g., advection, wind) considerations that could affect the derived surface temperatures.

## Limitations

The availability of satellite products is a key limitation. Of 21 LST scenes examined, only one scene was usable in that it lacked significant cloud cover and covered the study area geographically. The 16-day cycle on which Landsat images are acquired for a specific area does not afford researchers the opportunity to compare multiple images within a useful timeframe. Additionally, L5-TM data have a large spatial resolution (120 m), which might not capture the full heterogeneity of an urban environment (Johnson 2009).

Further, although we were able to match the time of acquisition of the ground data to the same time as the satellite data, the 1205 hours passing time of the Landsat satellite is not optimal for temperature–health studies. First, this time generally corresponds to a time of the day when ground temperatures transition from being cooler than air temperature to being warmer than air temperature. This means that, within the diurnal cycle, surface temperatures are not as significant drivers of air temperatures as they are later in the day. Second, exposure studies have tended to focus on maximum and minimum temperatures, and this time corresponds with neither of these (Basu 2009). Although shorter-term ground-based temperature timeframes did not yield strong correlations with SI or LST, composites of older satellite images could be one input into a more comprehensive planning tool or index to help describe vulnerability.

## Conclusions

Our results support the need for an increased effort, nationally, by public and private entities, to create useful remotely sensed data sources that can be applied to public health practice. A workshop report from the National Academy of Sciences (National Research Council 2007) discussed the challenges and potential applications of using remotely sensed data for public health. The report indicated that one of the major challenges to applying these remotely sensed data in the health arena is the limited *in situ* ground-truthing data accompanying remote sensing technology to verify analysis, and the high learning curve to using the tools required to analyze remotely sensed data. Our study gathered ground-truthing data needed to validate satellite-derived LST as well as SI. However, our results highlight that issues of spatial resolution, image availability over certain time periods, and the complex urban landscape remain challenges in the effort to integrate remotely sensed data with public health research and practice.

From a public health perspective, it is important to target resources and health interventions for the most vulnerable populations. The availability and usefulness of remote sensing data, integrated with social and economic demographic data, can provide a powerful tool for assessing vulnerability. A quality of life study conducted by Athens–Clarke County, Georgia, used one cloud-free image, aerial photographs, and U.S. Census data to overlay biophysical (land surface temperature, NDVI, land use, and land cover) and socioeconomic layers (population density, per capita income, median home value, education) to create a quality of life indicator (Lo and Faber 1997). The research found a strong relationship between biophysical and socioeconomic variables, which could be useful to assess vulnerability in the public health arena. In the field of heat epidemiology, being able to utilize a user-friendly data source such as SI as a proxy for surface temperature exposure can further our understanding of spatial vulnerability to heat. Another study has already shown that areas of the Detroit metropolitan region with high SI had statistically significant correlations with several sociodemographic variables: being ≥ 65 years of age and living alone, being able to leave the home, education level, living below the poverty line, and being nonwhite (White-Newsome et al. 2009).

There are several ways that remote sensing data could be better integrated into public health practice: *a*) increasing the capture frequency of remotely sensed images available for research and planning purposes; *b*) providing more highly processed data accessible to the public, at a finer resolution (10–15 m) and if possible at a higher temporal frequency that could be more useful for city and county level authorities; and *c*) commissioning more research to ground-truth satellite-derived land surface temperatures for different-sized urban areas, and establishing a set of fairly simple, standard best practices that can be used to estimate the influences of atmospheric and other factors on deriving a precise LST value from remote sensed imagery could be useful for planning for extreme heat events. Landsat data are the most consistent and widely available source of relatively high-resolution thermal information from satellites, but can be limited due to the number of clear images available at certain days and times. A gap in availability of these data exists, but continued acquisition of these data or data of comparable resolution has the potential to provide important spatial information about differential heat exposures.

One contribution of our study is to underscore the importance of the limitations of data, and emphasize that it is critical to have data that are accessible, useful, and timely for those working in public health. One of the main objectives of this study was to see whether a “non–remote-sensing professional” could create a tool—using available data—that can be used to estimate heat exposure. Reporting on the challenges we faced in doing this is one way to bring the issue to the attention of the remote-sensing community. As more practitioners demand these data, our research and other research that attempts to use the simplest methods should provide the impetus to fill the gaps in data to overcome these limitations.
